# Profile of Free and Conjugated Phenolic Compounds of Okra Pods Subjected to High-Humidity Hot-Air Impingement Blanching (HHAIB)

**DOI:** 10.3390/molecules30244665

**Published:** 2025-12-05

**Authors:** Danuta Zielińska, Natalia Płatosz, Kacper Górski, Magdalena Zielińska, Hong-Wei Xiao, Henryk Zieliński

**Affiliations:** 1 Department of Chemistry, University of Warmia and Mazury in Olsztyn, 10-721 Olsztyn, Poland; kacper.gorski@uwm.edu.pl; 2 Mass Spectrometry Laboratory, Institute of Animal Reproduction and Food Research, Polish Academy of Science, 10-638 Olsztyn, Poland; n.platosz@pan.olsztyn.pl; 3 Department of Electrical and Power Engineering, University of Warmia and Mazury in Olsztyn, 10-736 Olsztyn, Poland; m.zielinska@uwm.edu.pl; 4 College of Engineering, China Agricultural University, Beijing 100083, China; xhwcaugxy@163.com; 5 Team of Chemistry and Biodynamic of Food, Institute of Animal Reproduction and Food Research, Polish Academy of Science, 10-638 Olsztyn, Poland; h.zielinski@pan.olsztyn.pl

**Keywords:** high-humidity hot air impingement blanching (HHAIB), Okra pods, phenolic acids, flavonoids, micro-HPLC-QTRAP/MS/MS

## Abstract

The profiles of free, ester-bound, and glycoside-bound phenolic compounds in okra pods (OP) subjected to high-humidity hot-air impingement blanching (HHAIB) for up to 120 s were analyzed using a micro-HPLC-QTRAP/MS/MS method. Fourteen phenolic acids and ten flavonoids were identified in both unprocessed and HHAIB-treated samples. Phenolic acids and flavonoids were mainly present in free and ester-bound forms. HHAIB treatment up to 60 s resulted in a progressive decrease in the contents of free, ester-bound, and glycoside-bound phenolic acids. In contrast, the content of free flavonoids initially increased after 5 s of HHAIB, followed by a gradual decrease in conjugated forms with prolonged treatment. The results indicate that blanching for 90 s ensured the highest partial recovery of free and conjugated phenolic acids after initial loss as compared to the non-treated material. These findings demonstrate that HHAIB treatment can effectively modulate the phenolic composition of okra pods and help identify optimal processing conditions for maximizing the retention of bioactive compounds.

## 1. Introduction

Okra (*Abelmoschus esculentus* L. Moench) is an annual vegetable crop mainly cultivated in southern Europe, America, and tropical and subtropical regions of Asia [1]. It is valued for its edible green seed pods, which have long been used as a healthy vegetable and an important source of dietary therapeutics. Okra is recognized as a novel health-promoting vegetable with high nutritional value due to its wide range of bioactive compounds, including vitamins, dietary fiber, polysaccharides (pectins), pigments (chlorophyll-a and chlorophyll-b), and minerals [2,3]. Okra pods (OP) also contain volatile compounds, as reported by Ames and Macleod [4] and by Camciuc et al. [5].

Phenolic compounds, which include flavonoids and phenolic acids, exhibit highly diverse structures and properties and are very important secondary metabolites of okra pods due to their wide range of health-promoting functions, particularly their antioxidant activity [6]. From a nutritional perspective, these compounds may provide protection against the development of lifestyle-related diseases such as cancer, obesity, inflammation, diabetes, and cardiovascular or neurodegenerative diseases caused by oxidative and carbonyl stress [2,7,8,9].

Several compounds with potential antioxidant properties were identified in okra pods. Huang et al. [10] detected quercetin derivatives in okra, whereas Arapitsas [2] identified oligomeric catechins and flavonol derivatives in okra seeds, as well as hydroxycinnamic acid and quercetin derivatives in okra peels.

Since okra remains suitable for fresh consumption for only about a week or less after harvest, drying or dehydration can be applied to prevent spoilage and extend its shelf-life. Drying and dehydration are conventional processing methods that have been used since the early days of the food industry and are widely accepted by consumers [11]. In recent years, traditional drying techniques such as vacuum drying, convective drying, and sun drying have been applied to okra fruits. However, these methods are characterized by low drying rates, high consumption, and degradation of physicochemical properties [12,13].

Therefore, to improve the drying rate and reduce the negative effects of drying on the loss of physicochemical properties, it is crucial to apply an effective pretreatment method [14,15]. A common approach, such as hot water blanching, has been widely used [16]. However, to overcome some of its limitations, high-humidity hot-air impingement blanching (HHAIB) has recently been proposed as an alternative [17,18,19]. HHAIB extends the shelf-life of food products by inactivating polyphenol oxidase, enhancing phytochemical extraction, and mitigating the browning effect in food products [19].

It is well known that plants synthesize phenolic acids in both free and conjugated forms [20]. Phytochemicals, naturally occurring in plants in various forms, can generally be classified as compounds: free from chemical or physical interaction with other macromolecules; compounds physically entrapped within different cellular structures; compounds chemically bound to other macromolecules, such as pectins, fatty acids, and structural proteins; and compounds associated with insoluble material. Their thermal stability depends on factors such as temperature, pH, and the type and duration of processing. However, data on the qualitative identification of the polyphenolic profile of okra, including its free and conjugated forms, remain limited. Therefore, understanding the effect of HHAIB on changes in the phytochemical composition of okra pods is of great importance [21].

The aim of this study was (1) to investigate the profile of free and conjugated phenolic compounds in untreated okra pods (OP) and (2) to examine the effect of high-humidity hot-air impingement blanching (HHAIB) on their phytochemical profile. The study also aimed to determine how different blanching durations influence the retention and transformation of individual phenolic acids and flavonoids, providing insights into the optimal conditions for preserving bioactive compounds during processing. The novelty of this study is related to the simultaneous profiling of free, ester-bound, and glycoside-bound forms in okra pods subjected to HHAIB.

## 2. Results and Discussion

The profile and content of phenolic acids and flavonoids were determined using a highly sensitive micro-HPLC-QTRAP/MS/MS technique. Phenolic compounds were identified both as free forms and as conjugated forms released after acid and alkaline hydrolysis.

### 2.1. Profile and Content of Phenolic Acids in Okra Pods Subjected to HHAIB by Micro-HPLC-QTRAP/MS/MS

In this study, fourteen phenolic acids were identified in okra pods, including derivatives of hydroxycinnamic acid (ferulic, caffeic, sinapic, *para*-coumaric, chlorogenic, 3,4-dihydroxyhydrocinnamic and cynarin) and derivatives of hydroxybenzoic acid (protocatechuic, vanillic, homovanillic, salicylic, gentisic, syringic, p-hydroxybenzoic) were identified in okra pods (Table 1).

In the untreated okra pods (C—control), the dominant phenolic acids, considering both free and conjugated forms, were homovanillic, ferulic, p-coumaric, sinapic, vanillic, p-hydroxybenzoic, and protocatechuic acids (ranging from 366 to 134 µg/g dry matter). Caffeic and salicylic acids were present at approximately 100 µg/g dm, while gentisic and syringic acids ranged from 64 to 32 µg/g dm. Only trace amounts of chlorogenic and 3,4-dihydroxycinnamic acids were detected (below 0.24 µg/g dm) (Table 2).

The applied HHAIB treatment caused significant changes in the phenolic profile in okra pods. In the HHAIB-treated samples, the contents of sinapic, protocatechuic, and caffeic acids, evaluated in both free and conjugated forms, increased notably, with the highest levels observed after 90 s of blanching (Table 2). The contents of individual free, ester-bound, and glycoside-bound phenolic acids in untreated and HHAIB-treated okra pods are presented in Table 3a, 3b, and 3c, respectively.

The percentage distribution between free and conjugated forms (esters and glycosides) of individual phenolic acids and flavonoids in HHAIB-treated okra pods is presented in Table 4. The calculated proportion (%) between free and conjugated forms of phenolic acids showed that after HHAIB treatment, sinapic acid was present mainly in its free form, whereas caffeic and protocatechuic acids occurred predominantly in conjugated forms. No significant effect of HHAIB on the contents of gentisic and syringic acids was observed, and blanching for 90 s ensured the best stability of these compounds, which were present mainly in conjugated forms.

In contrast, the contents of homovanillic, ferulic, p-coumaric, vanillic, and salicylic acids decreased significantly, reaching a minimum after 60 s of blanching, followed by an increase, with the highest values observed after 90 s. These acids were found mainly in conjugated forms in the HHAIB-treated samples. The content of phenolic acids released from ester-bound conjugates was approximately four times higher than that of acids released from glycoside-bound conjugates (Table 4).

In summary, the total content of phenolic acids in okra pods blanched for 15, 30, 60, 90, and 120 s decreased by 20, 25, 31, 12, and 31%, respectively, compared with the total content in the control samples (Table 5). These results indicate that HHAIB treatment for 90 s provided the best retention of phenolic compounds. It should also be noted that after 90 s of blanching, ester-bound phenolic acids accounted for 61% of the total phenolic pool, whereas free and glycoside-bound forms represented only 24 and 16%, respectively (Table 5). The progressive decrease in the total content of phenolic acids was noted after HHAIB, and their highest partial recovery was observed after 90 s treatment.

### 2.2. Profile and Content of Flavonoids in Okra Pods Subjected to HHAIB by Micro-HPLC-QTRAP/MS/MS

In the untreated okra pod samples, ten flavonoids were identified (Table 1). The predominant flavonoids in total form were epicatechin, cynarin, quercetin, myricetin, and (−)epigallocatechin (ranging from 44 to 36 µg/g dm). The contents of verbascoside, orientin, and rutin varied between 10 and 5 µg/g dm, whereas vitexin, apigenin, and luteolin were less abundant (ranging from 0.49 to 0.02 µg/g dm) (Table 2). The contents of free, ester-bound, and glycoside-bound forms in untreated okra pods are presented in Table 3a, 3b, and 3c, respectively.

The applied HHAIB treatment caused noticeable changes in the flavonoid profile in okra pods (Table 3a–c). The contents of quercetin, rutin, and verbascoside in HHAIB-treated okra pods increased compared with the control sample. After 15 s of blanching, the quercetin content was approximately two times higher than in untreated pods, followed by a decrease up to 60 s, and then a maximum level was reached in the sample blanched for 120 s. Similar trends were observed for myricetin. In contrast, rutin content increased about fivefold after 30 s of blanching compared with the control, while longer blanching up to 120 s reduced its level to that of the untreated okra pods.

A significant increase of about 8–15 times in verbascoside content was recorded in HHAIB-treated okra pods. The contents of epicatechin and (−)epigallocatechin decreased by 17–30% and 36–77%, respectively, across the applied blanching times. In addition, a rapid reduction in cynarin and vitexin levels was observed after the first 15 s, while apigenin and luteolin contents remained very low after blanching, similar to those in the control sample.

The calculated proportion (%) between free and bound forms of identified flavonoids showed the presence of (−)epigallocatechin, verbascoside, orientin, apigenin, quercetin, rutin, and vitexin in untreated okra pods as aglycones, and these free forms also predominated after blanching. Epicatechin, myrecin, apigenin, and luteolin were present after blanching in both free and bound forms (Table 4). With increasing blanching time, the percentage of total flavonoids increased, whereas the total pool of phenolic acids decreased.

The total flavonoid content in okra pods, calculated as the sum of free, esterified, and glycoside-bound forms, initially increased by 58 and 29% after blanching for 15 s and 30 s of blanching, respectively, and then decreased by 19, 5, and 4% after 60, 90, and 120 s compared with the untreated sample (Table 5). These results indicate that 15 s of blanching is the most optimal duration for achieving the highest retention of total flavonoid content.

These findings are consistent with the results reported by Mounir et al. [22], who compared the swelling process with conventional shade drying of green okra pods. They observed an initial, transient increase in polyphenol content during processing, reaching a maximum, followed by a gradual decline.

However, it should be noted that while these trends agree with the observed changes in the results aligned with changes in profile of flavonoid profile, they contrast with the variations in the total phenolic acid content in okra pods subjected to HHAIB treatment.

In okra pods, total flavonoids and total phenolic acids contributed approximately 10 and 90%, respectively, to the overall phenolic pool, calculated as the sum of their free, esterified, and glycoside-bound forms (Table 5). Total phenolic compounds and flavonoids are the main contributors to the antioxidant capacity of okra, and significant differences among samples subjected to various drying methods have been reported previously [23]. The levels of these compounds and/or their derivatives in okra pods generally decrease after drying, with 45–89% of total phenolics and flavonoids retained, depending on the drying treatment.

Zielinska et al. [24] demonstrated the effect of HHAIB treatment on the total phenolic and flavonoid contents of okra pods, as determined by spectrophotometric assays. The present results are consistent with those observations, showing similar trends in the total contents of phenolic acids and flavonoids, calculated as the sum of their free, esterified, and glycoside-bound forms. However, it should be emphasized that the total phenolic content measured using the Folin–Ciocalteu reagent may be overestimated, since other reducing substances (e.g., reducing sugars) present in vegetables can also react with this reagent. Therefore, the application of the micro-HPLC-QTRAP/MS/MS technique provides more accurate and compound-specific quantification.

Data on the identification and quantification of phenolic compounds in okra pods remain limited. In our study, the fourteen phenolic acids and ten flavonoids were identified in both untreated and HHAIB-treated okra pods. Among the phenolic acids, the predominant compounds were homovanillic, ferulic, p-coumaric, sinapic, vanillic, p-hydroxybenzoic, and protocatechuic acids, whereas the dominant flavonoids were epicatechin, cynarin, quercetin, myricetin, and (−)epigallocatechin.

Recently, Meinhart et al. [25] identified chlorogenic and caffeic acids in three samples of Brazilian okra fruits using high-performance liquid chromatography (HPLC). The authors detected 3-*O*-caffeoylquinic, 5-*O*-caffeoylquinic, and 3,5-*O*-caffeoylquinic in the range of 1.25 to 25.5 µg/g fresh mass, which supports our findings. However, it should be noted that in our study, caffeic acid in okra pods was present mainly in the ester-bound form (Table 3b), and its content exceeded that of chlorogenic acid, which occurred only in the free form (Table 3a) by more than 350-fold.

The presence of sinapic acid and ferulic acid derivatives in okra pods and leaves was also reported by D’Urso et al. [26]. In okra pods, they identified several dominant phenolic acids, including 4-hydroxybenzoic acid, benzoic acid β-D-glucopyranosyl ester, tecomin, 3-methoxy benzoic acid, 3,4-dimethoxy benzoic acid, ferulic acid, 4-*O*-β-D-glucopyranoside, coumaric acid, sinapic acid, and 4-hydroxycinnamic acid. More recently, Mokgalabone et al. [27] detected n-O-caffeoyl glucaric acid, sinapoyl-glucaric acid, chlorogenic acid, and three isomers of ferulic acid in okra pods and leaves.

Regarding the flavonoid profile, only four quercetin derivatives had previously been isolated from okra and characterized, primarily in glycoside-bound forms, using NMR spectroscopy [10,28]. More recently, Arapitsas [2] identified oligomeric catechins and flavonol derivatives in okra seeds, whereas the polyphenolic profile of okra peels consisted mainly of hydroxycinnamic and quercetin derivatives. The identified hydroxycinnamic acid derivatives were primarily p-coumaric and sinapic acids, along with oligomeric catechins and both free and conjugated flavonols. Flavonols were found to be the major class of polyphenols present in okra seeds and peels.

In a pioneer study, Romdhane et al. [9] identified a total of eight flavonols using high-performance liquid chromatography coupled with diode array detection and electrospray ionization mass spectrometry (HPLC-DAD-MS/ESI), among which quercetin-3-*O*-glucoside was the predominant phenolic compound, followed by quercetin-*O*-pentosyl-hexoside and quercetin-dehexose.

In addition, three kaempferol derivatives were also detected. The RP-HPLC analysis conducted by Khomsug et al. [29] confirmed the presence of catechin, epicatechin, procyanidin B1, procyanidin B2, quercetin, and rutin in okra seeds and pulp.

It is also noteworthy to investigate the profile of free and conjugated phenolic compounds in okra stems, as this by-product of okra processing has been reported to contain significantly higher amounts of catechin derivatives, hydroxycinnamic acid derivatives, and flavonols compared with the levels found in okra seeds and peels [30].

Our findings are consistent with the results reported by D’Urso et al. [26] and Mokgalabone et al. [27] regarding the presence of quercetin, myricetin, and kaempferol glycosides in okra pods.

In our study, quercetin, rutin, epicatechin, and (−)epigallocatechin were found predominantly in their free forms, and to a lesser extent in ester-bound and glycoside-bound forms. For the first time, the presence of verbascoside, luteolin, apigenin, and their C-glucosides, namely vitexin and orientin, was confirmed. Moreover, the presence of both free and conjugated phenolic acids, such as vanillic, homovanillic, gentisic, caffeic, ferulic, and salicylic acids, was demonstrated for the first time, whereas chlorogenic acid was identified only in its free form.

The profile of free and conjugated phenolic compounds in okra pods appears to be strongly dependent on the cultivar. Wu et al. [31] reported the phenolic profile of five representative okra cultivars collected in China, identifying five individual phenolic compounds, including isoquercitrin, protocatechuic acid, quercetin-3-O-gentobioside, quercetin, and rutin, with isoquercitrin and quercetin-3-O-gentobioside as the major phenolics. In contrast, isoquercitrin was not detected in our study, which further supports the importance of okra cultivar variation, not only in terms of phenolic composition but also regarding nutritional value, free sugars, tocopherols, carotenoids, chlorophylls, and fatty acids, as described by Petropoulos et al. [8] for Mediterranean okra genotypes at different harvest stages. This study also revealed that ester-bound phenolic acids and flavonoid aglycones were relatively resistant to HHAIB, whereas free and glycoside-bound forms were less stable. In general, the total content of flavonoids was increased after HHAIB treatment up to 60 s, and the highest content was observed after 15 s of this treatment.

## 3. Materials and Methods

### 3.1. Materials

Okra (*Abelmoschus esculentus* cv. *Greenie*) pods were sourced from Yunnan Province, China, and purchased at a local market in Beijing, as described in detail by Zielinska et al. [24]. The calyx, pulp, and seeds were separated from whole okra pods. Only the pulp was used for processing, while the calyxes and seeds were discarded as waste. The okra pods were washed with tap water and blotted dry with filter paper. The prepared material was stored in a refrigerator at 4 ± 1 °C and 90% relative humidity for up to one week before processing. Prior to the experiments, the samples were cut into 3 cm pieces to ensure uniformity. The moisture content (MC) of okra pulp was determined according to AOAC: Official Method of Analysis [32]. The drying process was carried out for 24 h at 70 °C (13.3 kPa). The initial moisture content of fresh okra was 90% (wet basis).

### 3.2. Chemicals

Diethyl ether, trifluoroacetic acid (TFA), and MS-grade reagents, including acetonitrile, methanol (MeOH), water, and formic acid (FA), were purchased from Sigma-Aldrich (St. Louis, MO, USA). Pure flavonoid standards (vitexin, epigallocatechin, epicatechin, rutin, cynarin, verbascoside, myricetin, luteolin, quercetin, apigenin, and orientin) and phenolic acid standards (protocatechuic acid, chlorogenic acid, vanillic acid, p-hydroxybenzoic acid, 3,4-dihydroxyhydrocinnamic acid, gentisic acid, caffeic acid, homovanillic acid, syringic acid, p-coumaric acid, ferulic acid, sinapic acid, and salicylic acid) were obtained from Sigma Chemical Co. (St. Louis, MO, USA) and Extrasynthese (Genay, France).

### 3.3. High-Humidity Hot Air Impingement Blanching (HHAIB)

The HHAIB experiments were described in detail by Zielinska et al. [24]. The equipment consists of a series of round in-line nozzles, a steam generator to produce steam, an electric heater to heat the air, a centrifugal fan to supply the air flow and circulate the air flow, and a Proportional-Integral-Derivative (PID) controller (Omron, model E5CN, Tokyo, Japan) to control the blanching temperature. To apply HHAIB pretreatment, the steam generator was turned on, and when the impingement drying experiments were carried out, it was turned off. The trials were conducted at an air velocity of 14 m/s and a relative humidity of 35–40%. HHAIB pretreatment times were set to 15, 30, 60, 90, and 120 s. The temperature of the air in the jet was set at 110 °C. A single layer (mass of 100.00 ± 0.01 g) of 3 cm pieces (20 pieces of okra pods) was spread on a stainless-steel mesh in a working chamber when the temperature of the air reached a constant value of 110 °C. The change in sample mass was measured using an electronic balance (SP402; Ohaus Co., Parsippany, NJ, USA) with an accuracy of ±0.01 g. Untreated okra pods served as the control. Both control and blanched samples were preserved by freeze-drying at a constant shelf temperature of 10 °C, a cold trap temperature of −45 °C, and a vacuum level of 40–50 Pa (LGJ-10E; Ningbo Xinyl Ultrasonic Equipment Co., Ltd., Ningbo, China) [14]. Each HHAIB treatment was performed in three independent sample replicates following the experimental procedure.

### 3.4. Profile and Content of Phenolic Acids and Flavonoids in Okra Pods

#### 3.4.1. Extraction of Free, Ester, and Glycoside-Bound Forms of Phenolic Acids and Flavonoids

**Figure 1:**
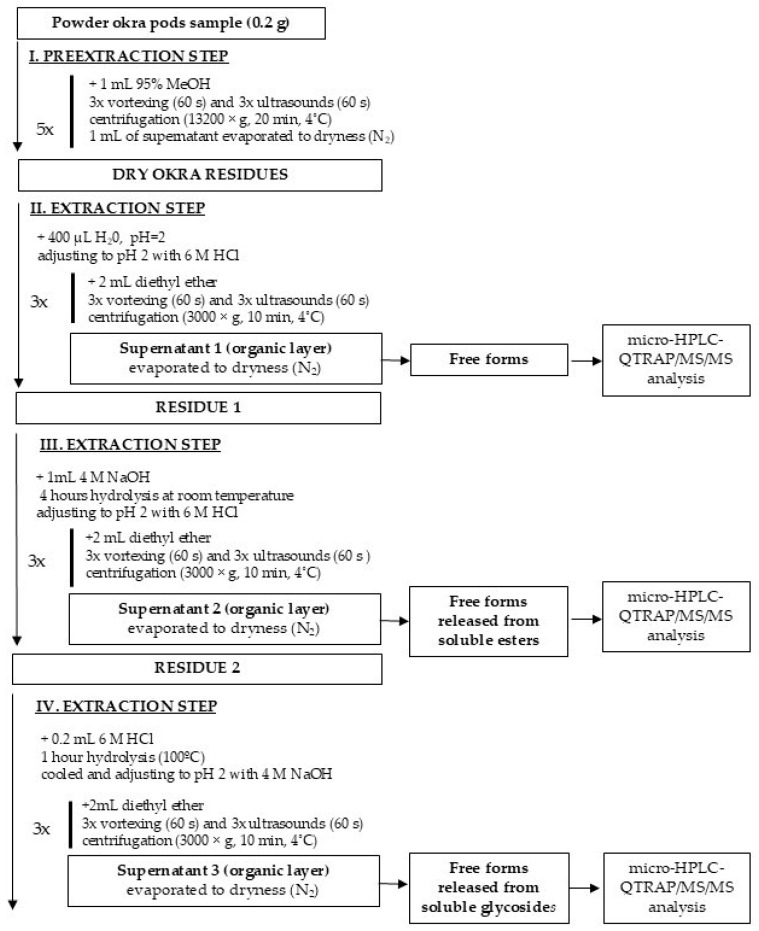
Experimental extraction scheme of free forms (Supernatant 1); ester-bound forms (Supernatant 2); and glycoside-bound forms (Supernatant 3) of phenolic acids and flavonoids.

The experimental scheme for the extraction of free, esterified, and glycoside-bound forms of phenolic acids and flavonoids from non-treated and HHAIB-treated okra pods is presented in Figure 1. A VC 750 sonificator (Sonics and Materials Inc., Newtown, CT, USA), a 5415R centrifuge (Eppendorf, Hamburg, Germany), and a heat block (Benchmark Scientific, Saryeville, NJ, USA) were used. The separated organic layer was collected, combined, and evaporated under a stream of nitrogen at 30 °C to remove any traces of diethyl ether. The dried extracts were stored at −80 °C until analysis.

The profile and content of phenolic compounds were analyzed according to the method described by Płatosz et al. [33]. The dried residues containing free phenolic compounds from okra pods, as well as those released from ester and glycoside bonds, were dissolved in 100 µL of 80% (*v*/*v*) methanol containing 0.95% (*v*/*v*) formic acid and centrifuged at 13,200× *g* for 20 min at 4 °C prior to analysis.

#### 3.4.2. Chromatographic Analysis

The analysis of free phenolic compounds from okra pods, as well as those related to ester and glycoside bonds, was performed using a micro-HPLC system (LC-200, Eksigent, Vanghan, ON, Canada) coupled with a QTRAP 5500 mass spectrometer (AB Sciex, Vaughan, ON, Canada). The system was equipped with a triple quadrupole, an ion trap, and an electrospray ionization (ESI) source. Optimal ESI-MS/MS conditions for the identification of phenolic compounds included settings for curtain gas, collision gas, ion spray voltage, temperature, ion source gases 1 and 2, declustering potential, entrance potential, collision energy, and collision cell exit potential. The HPLC systems and operating conditions are presented in Table 1. Identification and quantitation of phenolic acids and flavonoids were carried out by comparing their retention times and the presence of specific parent-product ion pairs in negative ion mode using the Multiple Reaction Monitoring (MRM) method, based on data obtained from authentic standards (Table 6) [24]. External calibration standards (0.01–0.5 µg/mL) exhibited linear calibration curves with a determination coefficient R^2^ ranging from 0.997 to 0.999. The results were expressed as µg per gram of okra dry matter (µg/g dm). All analyses were performed in triplicate for each sample.

### 3.5. Statistical Analysis

The calculations were performed using Statistica 12.0 software (TIBCO Software Inc., Palo Alto, CA, USA). The results were analyzed using one-way ANOVA and Duncan’s multiple range test at a significance level of *p* < 0.05. ANOVA analysis was performed for non-normally distributed data (*p* < 0.05). Statistically significant differences (*p* < 0.05) were indicated by different letters.

## 4. Conclusions

Fourteen phenolic acids were identified in both unprocessed and HHAIB-treated okra pods. The predominant acids were homovanillic, ferulic, *p*-coumaric, sinapic, vanillic, *p*-hydroxybenzoic, and protocatechuic acids, while epicatechin, cynarin, quercetin, myricetin, and (–)epigallocatechin were the dominant flavonoids. High-humidity hot-air impingement blanching (HHAIB) for up to 60 s resulted in a progressive decrease in free, ester-bound, and glycoside-bound phenolic acids. In contrast, free flavonoids initially increased after short HHAIB exposure (up to 15 s), followed by a gradual reduction in both glycoside- and ester-bound forms with longer treatment times. The findings demonstrate that both untreated and HHAIB-treated okra pods are rich sources of polyphenolic compounds, which occur mainly in free and ester-bound forms. For the first time, the presence of verbascoside, luteolin, apigenin, and their C-glucosides, namely vitexin and orientin, was confirmed.

These results, together with the high antioxidant potential of the identified phenolic acids and flavonoids, highlight the nutritional and functional importance of okra pods. Moreover, the application of micro-HPLC-QTRAP/MS/MS enabled highly sensitive and precise profiling of phytochemicals, providing valuable insights into the phenolic composition of okra and the effects of HHAIB treatment.

Future studies should focus on evaluating the bioaccessibility, bioavailability, and stability of these phenolic compounds during further processing and digestion, as well as on optimizing HHAIB parameters to maximize the retention of bioactive molecules in functional food applications.

Overall, the findings directly address the objectives of the study by characterizing the free and conjugated phenolic profiles of okra pods and elucidating how different HHAIB durations affect the retention and transformation of individual phenolic compounds.

## Figures and Tables

**Table 1:** Phenolic acids and flavonoids were identified in okra pods subjected to high-humidity hot-air impingement blanching.

No	Compounds	R_t_ (min)	[M-H]^-^ (*m*/*z*)	MS/MS (*m*/*z*)
***phenolic acids***				
**1**	protocatechuic acid	1.91	153	109/91
**2**	chlorogenic acid	2.21	353	191/161
**3**	vanillic acid	2.27	167	123/108/91
**4**	*p*-hydroxybenzoic acid	2.3	137	119/93/65
**5**	3,4-dihydroxyhydrocinnamic acid	2.31	181	137/75/59
**6**	cynarin	2.36	515	353/191/179
**7**	gentisic acid	2.41	153	135/109
**8**	caffeic acid	2.41	179	161/135/107
**9**	homovanillic acid	2.44	181	135/122
**10**	syringic acid	2.45	197	182/153/123
**11**	*p*-coumaric acid	2.51	163	145/119/93
**12**	ferulic acid	2.52	193	178/134/
**13**	sinapic acid	2.55	223	208/179/164
**14**	salicylic acid	2.79	137	119/93/65
***flavonoids***				
**15**	vitexin	1.92	431	323/311/283
**16**	epigallocatechin	2.01	305	165/125
**17**	epicatechin	2.33	289	245/203/109
**18**	rutin	2.34	609	463/301
**19**	verbascoside	2.46	623	461/161
**20**	myricetin	2.57	317	151/137
**21**	luteolin	2.71	285	151/133
**22**	quercetin	2.72	301	179/151
**23**	orientin	2.82	447	357/339/296
**24**	apigenin	3.00	269	225/151/117

**Table 2:** Content of free and conjugated (esters and glycosides) forms of phenolic acids and flavonoids in okra pods subjected to HHAIB.

	Phenolic Compounds	Contents [μg/g dm]					
		C	HHAIB 15 s	HHAIB 30 s	HHAIB 60 s	HHAIB 90 s	HHAIB 120 s
	***Phenolic acids***						
**1**	protocatechuic acid	134.70 ± 4.50 ^**cd**^	168.89 ± 15.05 ^**ab**^	110.30 ± 9.44 ^**d**^	137.45 ± 10.03 ^**cd**^	192.94 ± 15.97 ^**a**^	141.62 ± 1.28 ^**bc**^
**2**	chlorogenic acid	0.24 ± 0.00 ^**a**^	0.01 ± 0.00 ^**d**^	0.03 ± 0.00 ^**c**^	0.03 ± 0.00 ^**c**^	0.01 ± 0.00 ^**d**^	0.04 ± 0.00 ^**b**^
**3**	vanillic acid	185.88 ± 5.55 ^**a**^	60.16 ± 3.29 ^**b**^	50.07 ± 3.22 ^**c**^	36.30 ± 1.66 ^**d**^	57.88 ± 3.77 ^**bc**^	37.71 ± 1.78 ^**d**^
**4**	p-hydroxybenzoic acid	137.57 ± 5.81 ^**a**^	37.17 ± 3.63 ^**b**^	29.23 ± 1.96 ^**bc**^	23.36 ± 1.27 ^**c**^	35.77 ± 2.7 ^**b**^	29.19 ± 2.40 ^**bc**^
**5**	3.4-dihydroxyhydrocinnamic acid	0.24 ± 0.01 ^**a**^	0.12 ± 0.01 ^**d**^	0.20 ± 0.02 ^**b**^	0.17 ± 0.01 ^**bc**^	0.18 ± 0.01 ^**bc**^	0.15 ± 0.02 ^**cd**^
**6**	cynarin	39.92 ± 2.60 ^**a**^	0.87 ± 0.07 ^**b**^	nd	nd	nd	nd
**7**	gentisic acid	64.76 ± 2.81 ^**bc**^	73.15 ± 3.99 ^**ab**^	74.48 ± 4.92 ^**a**^	35.87 ± 0.35 ^**d**^	60.62 ± 2.79 ^**c**^	62.84 ± 4.11 ^**c**^
**8**	caffeic acid	104.22 ± 5.89 ^**c**^	164.55 ± 3.20 ^**b**^	178.45 ± 14.94 ^**b**^	189.50 ± 13.76 ^**b**^	367.16 ± 34.26 ^**a**^	107.38 ± 6.76 ^**c**^
**9**	homovanillic acid	366.41 ± 28.27 ^**a**^	300.44 ± 29.62 ^**b**^	297.15 ± 24.47 ^**b**^	244.11 ± 12.2 ^**bc**^	231.45 ± 14.98 ^**c**^	229.73 ± 18.28 ^**c**^
**10**	syringic acid	32.69 ± 1.37 ^**b**^	21.68 ± 1.60 ^**c**^	39.42 ± 2.41 ^**a**^	16.72 ± 1.59 ^**c**^	39.70 ± 1.08 ^**a**^	31.68 ± 2.81 ^**b**^
**11**	p-coumaric acid	265.44 ± 8.12 ^**a**^	224.56 ± 22.95 ^**ab**^	184.89 ± 14.73 ^**bc**^	174.44 ± 9.77 ^**c**^	199.79 ± 12.31 ^**bc**^	193.77 ± 19.42 ^**bc**^
**12**	ferulic acid	298.68 ± 6.38 ^**a**^	209.09 ± 14.82 ^**b**^	203.02 ± 15.73 ^**b**^	226.46 ± 20.44 ^**b**^	217.67 ± 21.81 ^**b**^	225.41 ± 5.90 ^**b**^
**13**	sinapic acid	189.40 ± 14.08 ^**b**^	228.82 ± 11.88 ^**a**^	220.83 ± 9.69 ^**ab**^	189.77 ± 18.10 ^**b**^	233.28 ± 13.82 ^**a**^	222.57 ± 13.03 ^**ab**^
**14**	salicylic acid	101.98 ± 6.21 ^**a**^	27.27 ± 1.94 ^**b**^	21.43 ± 1.33 ^**bc**^	17.46 ± 0.94 ^**c**^	27.07 ± 1.60 ^**b**^	21.76 ± 1.63 ^**bc**^
	***Flavonoids***						
**15**	vitexin	0.49 ± 0.04 ^**a**^	0.09 ± 0.01 ^**c**^	nd	0.14 ± 0.02 ^**bc**^	0.17 ± 0.03 ^**b**^	0.13 ± 0.01 ^**bc**^
**16**	(−)epigallocatechin	36.27 ± 2.27 ^**a**^	23.20 ± 2.75 ^**b**^	14.21 ± 0.44 ^**c**^	13.55 ± 0.53 ^**c**^	8.69 ± 0.75 ^**d**^	8.45 ± 0.72 ^**d**^
**17**	epicatechin	43.74 ± 3.02 ^**a**^	36.14 ± 2.26 ^**b**^	30.79 ± 1.13 ^**b**^	31.71 ± 0.98 ^**b**^	34.00 ± 1.71 ^**b**^	30.76 ± 2.02 ^**b**^
**18**	rutin	4.06 ± 0.35 ^**d**^	11.64 ± 1.21 ^**b**^	19.63 ± 0.99 ^**a**^	10.24 ± 1.10 ^**b**^	7.25 ± 0.36 ^**c**^	5.18 ± 0.14 ^**cd**^
**19**	verbascoside	10.31 ± 0.10 ^**d**^	152.09 ± 7.06 ^**a**^	120.32 ± 15.99 ^**b**^	72.75 ± 3.90 ^**c**^	88.23 ± 8.09 ^**c**^	83.35 ± 4.75 ^**c**^
**20**	myricetin	36.98 ± 2.79 ^**b**^	45.59 ± 1.70 ^**a**^	25.31 ± 1.57 ^**d**^	31.02 ± 1.31 ^**c**^	21.51 ± 1.39 ^**d**^	32.02 ± 2.49 ^**bc**^
**21**	luteolin	0.02 ± 0.01 ^**a**^	0.03 ± 0.01 ^**a**^	0.02 ± 0.01 ^**a**^	0.03 ± 0.01 ^**a**^	0.03 ± 0.01 ^**a**^	nd
**22**	quercetin	37.43 ± 1.25 ^**c**^	64.31 ± 4.02 ^**a**^	42.91 ± 1.12 ^**bc**^	27.98 ± 0.69 ^**d**^	38.33 ± 2.06 ^**c**^	44.67 ± 0.57 ^**b**^
**23**	orientin	4.64 ± 0.33 ^**b**^	4.9 ± 0.34 ^**ab**^	3.61 ± 0.16 ^**c**^	5.54 ± 0.36 ^**ab**^	5.83 ± 0.59 ^**a**^	2.71 ± 0.10 ^**c**^
**24**	apigenin	0.02 ± 0.01 ^**b**^	0.07 ± 0.01 ^**a**^	0.06 ± 0.01 ^**a**^	0.06 ± 0.01 ^**a**^	0.07 ± 0.01 ^**a**^	0.06 ± 0.01 ^**a**^

Table presents the values (mean ± standard deviation) averaged over three independent sample replicates. **^a,b,c,d^**—different letters in raw for each identified compound mean statistical differences between differently treated samples (*p* < 0.05). nd—not detected. Symbols: C—control sample (non-treated); HHAIB 15 s, 30 s, 60 s, 90 s and 120 s—okra pods subjected to high-humidity hot-air impingement blanching for 15, 30, 60, 90, and 120 s, respectively.

**Table 3:** (**a**). Content of free forms of phenolic acids and flavonoids in okra pods subjected to HHAIB. (**b**). Content of ester-bound phenolic acids and flavonoids in okra pods subjected to HHAIB. (**c**). Content of glycoside-bound phenolic acids and flavonoids in okra pods subjected to HHAIB.

	Phenolic Compounds	Contents [μg/g dm]					
		C	HHAIB 15 s	HHAIB 30 s	HHAIB 60 s	HHAIB 90 s	HHAIB 120 s
	**(a)**						
	***Phenolic acids***						
**1**	protocatechuic acid	5.95 ± 0.58 **^a^**	1.49 ± 0.09 **^b^**	1.03 ± 0.02 **^b^**	0.99 ± 0.02 **^b^**	1.01 ± 0.13 **^b^**	1.14 ± 0.03 **^b^**
**2**	chlorogenic acid	0.24 ± 0.00 **^a^**	0.01 ± 0.00 **^b^**	0.03 ± 0.0 **^b^**	0.03 ± 0.00 **^b^**	0.01 ± 0.0 **^b^**	0.04 ± 0.00 **^b^**
**3**	vanillic acid	146.06 ± 2.91 **^a^**	12.51 ± 1.02 **^cd^**	16.37 ± 1.05 **^b^**	11.17 ± 0.39 **^d^**	12.08 ± 0.56 **^c^**	9.82 ± 0.41 **^e^**
**4**	p-hydroxybenzoic acid	86.35 ± 4.46 **^a^**	12.55 ± 1.56 **^b^**	8.97 ± 0.47 **^d^**	10.24 ± 0.38 **^c^**	11.60 ± 1.08^**bc**^	10.11 ± 0.81 **^c^**
**5**	3,4-dihydroxyhydrocinnamic acid	0.09 ± 0.00	nd	nd	nd	nd	nd
**6**	cynarin	17.25 ± 1.26	nd	nd	nd	nd	nd
**7**	gentisic acid	15.15 ± 0.40 **^d^**	34.26 ± 1.68 **^b^**	44.95 ± 4.36 **^a^**	15.28 ± 0.06 **^d^**	24.25 ± 0.95 **^c^**	36.86 ± 2.46 **^b^**
**8**	caffeic acid	11.54 ± 0.46 **^b^**	5.14 ± 0.26**^e^**	8.80 ± 0.47 **^d^**	10.00 ± 0.30 **^c^**	19.21 ± 1.77 **^a^**	18.20 ± 1.74 **^a^**
**9**	homovanillic acid	138.07 ± 9.98 **^a^**	108.10 ± 9.35 **^b^**	122.08 ± 5.89 **^b^**	94.18 ± 5.37 **^c^**	83.74 ± 8.34 **^cd^**	73.48 ± 7.52 **^d^**
**10**	syringic acid	3.66 ± 0.20 **^c^**	5.77 ± 0.53 **^b^**	6.26 ± 0.39 **^b^**	3.93 ± 0.49 **^c^**	9.50 ± 0.25 **^a^**	2.50 ± 0.37 **^d^**
**11**	p-coumaric acid	35.48 ± 2.71 **^a^**	29.13 ± 2.12 **^b^**	18.82 ± 1.88 **^d^**	16.36 ± 0.88 **^d^**	21.74 ± 1.67 **^c^**	18.21 ± 1.60 **^d^**
**12**	ferulic acid	122.05 ± 0.92 **^a^**	48.66 ± 2.07 **^c^**	54.79 ± 1.60 **^b^**	51.47 ± 5.72 **^bc^**	53.76 ± 4.65 **^bc^**	56.83 ± 2.73 **^b^**
**13**	sinapic acid	110.32 ± 8.06 **^d^**	169.71 ± 7.04 **^a^**	149.28 ± 7.51**^b^**	138.42 ± 13.26 **^bc^**	145.38 ± 9.03**^b^**	119.51 ± 7.14 **^cd^**
**14**	salicylic acid	63.57 ± 5.20 **^a^**	8.31 ± 0.75 **^b^**	6.72 ± 0.35 **^c^**	7.68 ± 0.28 **^b^**	8.70 ± 0.81 **^b^**	7.58 ± 0.61 **^c^**
	***Flavonoids***						
**15**	vitexin	0.38 ± 0.03 **^a^**	0.09 ± 0.01 **^b^**	nd	0.14 ± 0.02 **^b^**	0.17 ± 0.03 **^b^**	0.13 ± 0.01 **^b^**
**16**	(−)epigallocatechin	36.27 ± 2.27 **^a^**	23.20 ± 2.75 **^b^**	14.21 ± 0.44 **^c^**	13.55 ± 0.53 **^c^**	8.69 ± 0.75 **^d^**	8.45 ± 0.72**^d^**
**17**	epicatechin	22.28 ± 1.40 **^a^**	18.33 ± 1.92 **^a^**	14.85 ± 0.88 **^b^**	18.80 ± 0.47 **^a^**	19.99 ± 2.09 **^a^**	19.68 ± 1.42 **^a^**
**18**	rutin	2.85 ± 0.25 **^f^**	11.10 ± 1.17 **^b^**	17.86 ± 0.89 **^a^**	9.11 ± 0.94 **^c^**	5.67 ± 0.26 **^d^**	4.45 ± 0.10 **^e^**
**19**	verbascoside	10.31 ± 0.10 **^f^**	152.09 ± 7.06 **^b^**	120.32 ± 15.99 **^a^**	72.75 ± 3.90 **^e^**	88.23 ± 8.09 **^d^**	83.35 ± 4.75 **^c^**
**20**	myricetin	7.76 ± 0.56 **^c^**	36.97 ± 1.18 **^a^**	15.24 ± 0.78 **^b^**	15.46 ± 1.02 **^b^**	15.38 ± 1.18 **^b^**	16.99 ± 1.49 **^b^**
**21**	luteolin	0.01 ± 0.01 **^a^**	0.02 ± 0.01 **^a^**	0.01 ± 0.01 **^a^**	0.02 ± 0.01 **^a^**	0.02 ± 0.01 **^a^**	nd
**22**	quercetin	34.01 ± 1.10 **^c^**	63.67 ± 3.97 **^a^**	41.10 ± 1.10 **^b^**	26.53 ± 0.58 **^d^**	34.58 ± 1.79 **^c^**	39.73 ± 0.35 **^b^**
**23**	orientin	4.64 ± 0.33 **^a^**	3.00 ± 0.26 **^c^**	2.22 ± 0.11 **^e^**	3.73 ± 0.26 **^b^**	3.41 ± 0.38 **^b^**	2.71 ± 0.02 **^d^**
**24**	apigenin	0.02 ± 0.01 **^a^**	0.02 ± 0.01 **^a^**	0.02 ± 0.01 **^a^**	0.03 ± 0.01 **^a^**	0.02 ± 0.01 **^a^**	0.03 ± 0.01 **^a^**
	***Total phenolic acids and flavonoids***	874.32 ± 43.20 **^a^**	744.13 ± 44.81 **^b^**	663.93 ± 44.20 **^bc^**	519.87 ± 34.89 **^d^**	567.14 ± 43.83 **^cd^**	529.80 ± 34.29 **^d^**
**(b)**							
	***Phenolic acids***						
**1**	protocatechuic acid	16.58 ± 0.87 **^a^**	16.40 ± 1.24 **^a^**	10.35 ± 0.82 **^c^**	12.23 ± 1.35 **^b^**	13.62 ± 1.02 **^b^**	11.61 ± 1.36 **^b^**
**2**	chlorogenic acid	nd	nd	nd	nd	nd	nd
**3**	vanillic acid	19.40 ± 0.93 **^c^**	28.99 ± 1.70 **^a^**	26.45 ± 1.55 **^ab^**	20.20 ± 0.83 **^c^**	25.11 ± 1.23 **^b^**	12.31 ± 0.89 **^d^**
**4**	p-hydroxybenzoic acid	10.34 ± 0.64 **^b^**	10.89 ± 0.99 **^b^**	11.08 ± 0.62 **^b^**	12.38 ± 0.83 **^ab^**	13.55 ± 0.71 **^a^**	13.05 ± 0.92 **^a^**
**5**	3,4-dihydroxyhydrocinnamic acid	0.15 ± 0.01 **^c^**	0.12 ± 0.01 **^d^**	0.20 ± 0.02 **^a^**	0.17 ± 0.01 **^ab^**	0.18 ± 0.01 **^ab^**	0.15 ± 0.02 **^bcd^**
**6**	cynarin	22.67 ± 1.34 **^a^**	0.87 ± 0.07 **^b^**	nd	nd	nd	nd
**7**	gentisic acid	32.75 ± 0.81 **^a^**	20.19 ± 0.60 **^c^**	20.49 ± 0.34 **^c^**	20.59 ± 0.29 **^c^**	23.00 ± 0.78 **^b^**	21.10 ± 1.18 **^bc^**
**8**	caffeic acid	84.61 ± 4.87 **^d^**	154.11 ± 2.92 **^c^**	164.47 ± 14.09 **^bc^**	179.50 ± 13.46 **^b^**	347.95 ± 32.49 **^a^**	89.18 ± 5.02 **^d^**
**9**	homovanillic acid	203.81 ± 17.07 **^a^**	192.34 ± 20.27 **^a^**	175.07 ± 18.58 **^a^**	149.93 ± 6.83 **^b^**	147.71 ± 6.64 **^b^**	156.25 ± 10.76 **^b^**
**10**	syringic acid	14.99 ± 0.54 **^c^**	10.81 ± 0.75 **^d^**	19.79 ± 1.01 **^b^**	10.62 ± 0.96 **^d^**	20.13 ± 0.37 **^b^**	24.13 ± 2.35 **^a^**
**11**	p-coumaric acid	223.81 ± 4.99 **^a^**	192.79 ± 20.55 **^b^**	159.91 ± 12.34 **^bc^**	154.58 ± 8.58 **^c^**	174.53 ± 10.28 **^bc^**	169.63 ± 17.23 **^bc^**
**12**	ferulic acid	170.43 ± 4.84 **^a^**	157.04 ± 12.45 **^a^**	145.92 ± 13.86 **^a^**	173.38 ± 14.58 **^a^**	161.03 ± 16.86 **^a^**	165.85 ± 2.92 **^a^**
**13**	sinapic acid	60.29 ± 5.28 **^c^**	51.70 ± 4.61 **^d^**	65.23 ± 1.58 **^c^**	50.02 ± 4.81 **^d^**	82.70 ± 4.42 **^b^**	95.71 ± 5.61 **^a^**
**14**	salicylic acid	7.75 ± 0.48 **^c^**	7.80 ± 0.34 **^c^**	8.31 ± 0.47 **^c^**	9.29 ± 0.62 **^bc^**	10.41 ± 0.11 **^a^**	9.78 ± 0.69 **^ab^**
	***Flavonoids***						
**15**	vitexin	0.11 ± 0.01	nd	nd	nd	nd	nd
**16**	(−)epigallocatechin	nd	nd	nd	nd	nd	nd
**17**	epicatechin	12.75 ± 1.13 **^a^**	9.21 ± 0.21 **^b^**	6.75 ± 0.28 **^d^**	9.63 ± 0.16 **^b^**	9.95 ± 0.74 **^c^**	7.67 ± 0.34 **^c^**
**18**	rutin	1.21 ± 0.10 **^b^**	0.54 ± 0.04 **^d^**	1.77 ± 0.10 **^a^**	1.13 ± 0.16 **^b^**	1.58 ± 0.10 **^a^**	0.73 ± 0.04 **^c^**
**19**	verbascoside	nd	nd	nd	nd	nd	nd
**20**	myricetin	25.33 ± 1.88 **^a^**	8.62 ± 0.52 **^d^**	10.07 ± 0.79 **^c^**	15.56 ± 0.59 **^b^**	5.84 ± 0.18 **^e^**	14.78 ± 0.98 **^b^**
**21**	luteolin	0.01 ± 0.00 **^a^**	0.01 ± 0.00 **^a^**	0.01 ± 0.00 **^a^**	0.01 ± 0.00 **^a^**	0.01 ± 0.00 **^a^**	nd
**22**	quercetin	2.44 ± 0.10 **^c^**	0.51 ± 0.04 **^f^**	1.75 ± 0.02 **^d^**	1.44 ± 0.11 **^e^**	3.33 ± 0.25 **^b^**	4.39 ± 0.17 **^a^**
**23**	orientin	nd	1.90 ± 0.08 **^b^**	1.39 ± 0.05 **^c^**	1.81 ± 0.10 **^b^**	2.42 ± 0.21 **^a^**	nd
**24**	apigenin	nd	nd	nd	nd	nd	nd
	***Total phenolic acids and flavonoids***	909.43 ± 45.89 **^bc^**	864.84 ± 67.39 **^c^**	829.01 ± 66.52 **^c^**	822.47 ± 54.27 **^c^**	1043.05 ± 76.40 **^a^**	796.32 ± 50.48 **^c^**
**(c)**							
	***Phenolic acids***						
**1**	protocatechuic acid	112.17 ± 3.05 **^d^**	151.00 ± 13.72 **^b^**	98.92 ± 8.60 **^d^**	124.23 ± 8.66 **^c^**	178.31 ± 14.82 **^a^**	128.87 ± 8.89 **^c^**
**2**	chlorogenic acid	nd	nd	nd	nd	nd	nd
**3**	vanillic acid	20.42 ± 1.71 **^a^**	18.66 ± 0.57 **^a^**	7.25 ± 0.62 **^c^**	4.93 ± 0.44 **^d^**	20.69 ± 1.98 **^a^**	15.58 ± 0.48 **^b^**
**4**	p-hydroxybenzoic acid	40.88 ± 0.71 **^a^**	13.73 ± 1.08 **^b^**	9.18 ± 0.87 **^c^**	0.74 ± 0.06 **^e^**	10.62 ± 0.91^**c**^	6.03 ± 0.67 **^d^**
**5**	3.4-dihydroxyhydrocinnamic acid	nd	nd	nd	nd	nd	nd
**6**	cynarin	nd	nd	nd	nd	nd	nd
**7**	gentisic acid	16.86 ± 1.60 **^a^**	18.70 ± 1.71 **^a^**	9.04 ± 0.22 **^c^**	nd	13.37 ± 1.06 **^b^**	4.88 ± 0.47 **^d^**
**8**	caffeic acid	8.07 ± 0.56 **^a^**	5.30 ± 0.02**^b^**	5.18 ± 0.38 **^b^**	nd	nd	nd
**9**	homovanillic acid	24.53 ± 1.22	nd	nd	nd	nd	nd
**10**	syringic acid	14.04 ± 0.63 **^a^**	5.10 ± 0.32 **^c^**	13.37 ± 1.01 **^a^**	2.17 ± 0.14 **^d^**	10.07 ± 0.46 **^b^**	5.05 ± 0.09 **^c^**
**11**	p-coumaric acid	6.15 ± 0.42 **^a^**	2.64 ± 0.28 **^c^**	6.16 ± 0.51 **^a^**	3.50 ± 0.31 **^b^**	3.52 ± 0.36 **^b^**	5.93 ± 0.59 **^a^**
**12**	ferulic acid	6.20 ± 0.62 **^a^**	3.39 ± 0.30 **^b^**	2.31 ± 0.27 **^d^**	1.61 ± 0.14 **^e^**	2.88 ± 0.30 **^bc^**	2.73 ± 0.25 **^c^**
**13**	sinapic acid	18.79 ± 0.74 **^a^**	7.41 ± 0.23 **^b^**	6.32 ± 0.60 **^c^**	1.33 ± 0.03 **^e^**	5.20 ± 0.37 **^d^**	7.35 ± 0.28 **^b^**
**14**	salicylic acid	30.66 ± 0.53 **^a^**	11.16 ± 0.85 **^b^**	6.40 ± 0.51 **^d^**	0.49 ± 0.04 **^f^**	7.96 ± 0.68 **^c^**	4.40 ± 0.33 **^e^**
	***Flavonoids***						
**15**	vitexin	nd	nd	nd	nd	nd	nd
**16**	(−)epigallocatechin	nd	nd	nd	nd	nd	nd
**17**	epicatechin	8.71 ± 0.49 **^ab^**	8.60 ± 0.13 **^b^**	9.19 ± 0.27 **^a^**	3.28 ± 0.35 **^d^**	4.06 ± 0.22 **^c^**	3.41 ± 0.26 **^d^**
**18**	rutin	nd	nd	nd	nd	nd	nd
**19**	verbascoside	nd	nd	nd	nd	nd	nd
**20**	myricetin	3.89 ± 0.35 **^a^**	nd	nd	nd	0.29 ± 0.03 **^b^**	0.25 ± 0.02 **^b^**
**21**	luteolin	nd	nd	nd	nd	nd	nd
**22**	quercetin	0.98 ± 0.05 **^a^**	0.13 ± 0.01 **^d^**	0.06 ± 0.00 **^e^**	0.01 ± 0.00 **^f^**	0.42 ± 0.02 **^c^**	0.55 ± 0.05 **^b^**
**23**	orientin	nd	nd	nd	nd	nd	nd
**24**	apigenin	nd	0.05 ± 0.00 **^a^**	0.04 ± 0.00 **^a^**	0.03 ± 0.00 **^a^**	0.05 ± 0.00 **^a^**	0.03 ± 0.00 **^a^**
	***Total phenolic acids and flavonoids***	312.35 ± 12.68 **^a^**	245.87 ± 19.22 **^b^**	173.42 ± 0.27 **^cd^**	142.32 ± 10.17 **^d^**	257.44 ± 21.21 **^b^**	185.06 ± 12.38 **^c^**

Table presents the values (mean ± standard deviation) averaged over three independent sample replicates. **^a,b,c,d,e,f^**—different letters in raw for each identified compound mean statistical differences between differently treated samples (*p* < 0.05). nd—not detected. Symbols: C—control sample (non-treated); HHAIB 15 s, 30 s, 60 s, 90 s, and 120 s—okra pods subjected to high-humidity hot-air impingement blanching for 15, 30, 60, 90, and 120 s, respectively.

**Table 4:** Partition (%) between free and conjugated (esters and glycosides) forms of individual phenolic acids and flavonoids in okra pods subjected to HHAIB.

Phenolic Compounds	C		HHAIB 15 s		HHAIB 30 s		HHAIB 60 s		HHAIB 90 s		HHAIB 120 s	
	Free	Conj.	Free	Conj.	Free	Conj.	Free	Conj.	Free	Conj.	Free	Conj.
***Phenolic acids***												
protocatechuic acid	5	95	1	99	1	99	1	99	1	99	1	99
chlorogenic acid	100	0	100	0	100	0	100	0	100	0	100	0
vanillic acid	78	22	21	79	33	67	30	70	21	79	26	74
p-hydroxybenzoic acid	63	37	34	66	31	69	44	56	33	67	35	65
3.4-dihydroxyhydrocinnamic acid	37	63	0	100	0	100	0	100	0	100	0	100
cynarin	43	57	nd	nd	nd	nd	nd	nd	nd	nd	nd	nd
gentisic acid	23	73	47	53	60	40	43	57	40	60	59	41
caffeic acid	11	89	3	97	5	95	5	95	5	95	17	83
homovanillic acid	38	62	36	64	41	59	39	61	36	64	32	68
syringic acid	11	89	27	73	16	84	23	77	24	76	8	92
p-coumaric acid	13	87	13	87	10	90	10	90	11	89	10	90
ferulic acid	41	59	23	77	27	73	23	77	25	75	25	75
sinapic acid	58	42	74	26	68	32	73	27	62	28	54	46
salicylic acid	62	38	30	70	31	69	44	56	32	68	35	65
***Flavonoids***												
vitexin	76	24	100	0	nd	nd	100	0	100	0	100	0
rutin	70	30	95	5	91	9	89	11	78	22	86	14
(−)epigallocatechin	100	0	100	0	100	0	100	0	100	0	100	0
myricetin	21	79	81	29	60	40	50	50	71	29	53	47
verbascoside	100	0	100	0	100	0	100	0	100	0	100	0
epicatechin	51	49	51	49	48	52	59	41	59	41	64	26
quercetin	91	9	99	1	96	4	95	5	90	10	89	11
luteolin	50	50	67	33	50	50	67	33	67	33	nd	nd
orientin	100	0	61	39	62	38	67	33	58	42	100	0
apigenin	100	0	28	72	33	67	50	50	28	72	50	50

Symbols: C—control sample (non-treated); HHAIB 15 s, 30 s, 60 s, 90 s, and 120 s—okra pods subjected to high-humidity hot-air impingement blanching for 15, 30, 60, 90, and 120 s, respectively; nd—not determined.

**Table 5:** The total content of phenolic acids and flavonoids in okra pods subjected to HHAIB treatment is the sum of their free, ester, and glycoside forms.

Phenolic Compounds	Contents [μg/g dm]					
	C	HHAIB 15 s	HHAIB 30 s	HHAIB 60 s	HHAIB 90 s	HHAIB 120 s
Phenolic acids (free form)	755.78 ± 38.79 **^a^**	435.64 ± 26.47 **^bc^**	438.10 ± 23.99 **^b^**	359.75 ± 27.15 ^cd^	390.98 ± 29.24 **^bcd^**	354.28 ± 25.42 **^d^**
Phenolic acids (ester bound)	867.58 ± 42.67 **^b^**	844.05 ± 66.50 **^b^**	807.27 ± 65.28 **^b^**	792.89 ± 53.15 **^b^**	1019.92 ± 74.92 **^a^**	768.75 ± 48.95 **^b^**
Phenolic acids (glycoside bound)	298.77 ± 11.79 **^a^**	237.09 ± 19.08 **^b^**	164.13 ± 13.59 **^cd^**	139.00 ± 9.82 **^d^**	252.62 ± 20.94 **^b^**	180.82 ± 12.05 **^c^**
**Total content of phenolic acids**	**1922.13 ± 93.25 ^a^**	**1516.78 ± 112.05 ^bc^**	**1409.50 ± 102.86 ^bc^**	**1291.64 ± 90.12 ^c^**	**1663.52 ± 125.10 ^ab^**	**1303.85 ± 86.42 ^c^**
Flavonoids (free form)	118.54 ± 6.06 **^d^**	308.49 ± 18.34 **^a^**	225.83 ± 20.21 **^b^**	160.12 ± 7.74 **^cd^**	176.16 ± 14.59 **^c^**	175.52 ± 8.87 **^c^**
Flavonoids (ester bound)	41.85 ± 3.22 **^a^**	20.79 ± 0.89 **^c^**	21.74 ± 1.24 **^c^**	29.58 ± 1.12 **^b^**	23.13 ± 1.48 **^c^**	27.57 ± 1.53 **^bc^**
Flavonoids (glycoside bound)	13.58 ± 0.89 **^a^**	8.78 ± 0.14 **^b^**	9.29 ± 0.27 **^b^**	3.32 ± 0.35 **^d^**	4.82 ± 0.35 **^c^**	4.24 ± 0.33 **^cd^**
**Total content of flavonoids**	**173.97 ± 10.17 ^bc^**	**338.06 ± 19.37 ^a^**	**256.86 ± 21.72 ^b^**	**193.02 ± 9.21 ^c^**	**204.11 ± 16.42 ^c^**	**207.33 ± 10.73 ^c^**
**Total content of phenolic acids and flavonoids**	**2096.10 ± 103.42 ^a^**	**1854.84 ± 131.42 ^ab^**	**1666.36 ± 124.58 ^bc^**	**1484.66 ± 99.33 ^c^**	**1867.63 ± 141.52 ^ab^**	**1511.18 ± 97.15 ^c^**

Table presents the values (mean ± standard deviation) averaged over 3 independent sample replicates. **^a,b,c,d^**—different letters in raw for each identified compound mean statistical differences between differently treated samples (*p* < 0.05). Symbols: C—control sample (non-treated); HHAIB 15 s, 30 s, 60 s, 90 s, and 120 s—okra pods subjected to high-humidity hot air impingement blanching for 15, 30, 60, 90, and 120 s, respectively.

**Table 6:** The micro-HPLC-QTRAP/MS/MS system and conditions.

Apparatus	LC-200, Eksigent, Vanghan, ON, Canada
Detector	Mass spectrometer QTRAP 5500, AB Sciex, Vaughan, ON, Canada
Column	HALO C18 column (50 mm × 0.5 mm, 2.7 µm), Eksigent, Vaughan, ON, Canada
Mobile phase	Solvent A (water/formic acid; 99.05/0.95; *v*/*v*) Solvent B (acetonitrile/formic acid, 99.05/0.95, *v*/*v*)
Gradient program	5% B (0–0.5 min) 5% to 90% B (0.5–2 min) 90% B (2–2.5 min) 90% to 5% B (2.5–2.7 min) 5% B (3 min)
Optical ESI-MS/MS conditions	Negative ionization, curtain gas: 20 L/min; collision gas: 9 L/min; ion spray voltage: −5300 V; temperature: 350 °C; 1 ion source gas: 35 L/min; 2 ion source gas: 30 L/min; declastering potential: 100 V; entrance potential: 10 V; collision energy: 40 eV; collision cell exit potential: 20 V
Injection volume	5 µL
Flow rate	15 µL/min
Column temperature	45 °C

## Data Availability

The original contributions presented in the study are included in the article; further inquiries can be directed to the corresponding author.
